# IGF1R levels in the brain negatively correlate with longevity in 16 rodent species

**DOI:** 10.18632/aging.100552

**Published:** 2013-04-25

**Authors:** Jorge Azpurua, Jiang-Nan Yang, Michael Van Meter, Zhengshan Liu, Julie Kim, Aliny AB Lobo Ladd, Antonio Augusto Coppi, Vera Gorbunova, Andrei Seluanov

**Affiliations:** ^1^ Department of Biology, University of Rochester, Rochester NY 14627, USA; ^2^ Laboratory of Stochastic Stereology and Chemical Anatomy, Department of Surgery, College of Veterinary Medicine, University of São Paulo, São Paulo, Brazil

**Keywords:** aging, IGF1 receptor, rodents, comparative approach, life span

## Abstract

The insulin/insulin-like growth factor signaling (IIS) pathway is a major conserved regulator of aging. Nematode, fruit fly and mouse mutants with reduced IIS signaling exhibit extended lifespan. These mutants are often dwarfs leading to the idea that small body mass correlates with longevity within species. However, when different species are compared, larger animals are typically longer-lived. Hence, the role of IIS in the evolution of life history traits remains unresolved. Here we used comparative approach to test whether IGF1R signaling changes in response to selection on lifespan or body mass and whether specific tissues are involved. The IGF1R levels in the heart, lungs, kidneys, and brains of sixteen rodent species with highly diverse lifespans and body masses were measured via immunoblot after epitope conservation analysis. We report that IGF1R levels display strong negative correlation with maximum lifespan only in brain tissue and no significant correlations with body mass for any organ. The brain-IGF1R and lifespan correlation holds when phylogenetic non-independence of data-points is taken into account. These results suggest that modulation of IGF1R signaling in nervous tissue, but not in the peripheral tissues, is an important factor in the evolution of longevity in mammals.

## INTRODUCTION

A major discovery in the field of aging research is that large differences in maximum lifespan can be attributed to the effects of a few major signaling pathways. The first ‘aging gene' was discovered in the nematode worm, *C. elegans*, in a genetic screen for long-lived mutants [1] and later identified as a homolog of the insulin receptor [2]. Similarly, in *Drosophila*, perturbation of the *INR* gene extended lifespan by up to 85% [3]. In both systems, the lifespan phenotype was dependent on insulin/IGF1 signaling (hereafter IIS) mediated inhibition of the *daf-16*/*FOXO* transcription factor [4] which is involved in diverse processes such as stress response, immunity and metabolism.

In mammals, the ancestral insulin receptor gene was duplicated and diverged into *INSR* and *IGF1R*. Both insulin and IGF1 seem to play a role in mammalian aging, since mouse strains with reduced IIS show lifespan extension [5]. Because serum IGF1 secretion from the liver is dependent on growth hormone signaling [6] Snell and Ames dwarf mice are long-lived [7]. High plasma IGF1 levels show an inverse correlation with median lifespan [8]. Growth hormone receptor knock-out (GHRKO) mice are also long-lived [9]. IGF1R heterozygous knock-out mice showed a 26% average lifespan extension [10] although attempts to replicate the experiment with a larger sample size failed [11]. In humans, reduced IGF1 signaling has been implicated in life extension by showing that a less active form of the protein is enriched in centenarians [12]. Furthermore, the offspring of human centenarians have low circulating levels of IGF1 [13].

The evolutionary relationships between longevity, body mass, and IIS is not clear. Within species, smaller size is correlated to longer lifespans [14-16]. Within species, lowered IIS signaling is also correlated to longer lifespan [2, 10, 12]. However, across species, longer lifespans are correlated with larger body mass. Therefore, if IIS is predominantly being selected for increased body mass across taxa, then one might expect longer-lived species to actually have higher levels of IIS than shorter-lived taxa.

The potent effect of IGF1 signaling on the body mass of mammals has previously been demonstrated in dogs, where a single IGF1 allele was shown to be the main determinant of small size in dogs [17]. However, a recent comparative study found a negative correlation to body mass in a cross-taxa comparison of the plasma IGF1 levels in mammals [18]. Because the body mass range of Rodentia varies by multiple orders of magnitude and lifespan ranges from 3 to 31 years, it is possible to compare the effects of IGF1 signaling on lifespan and body mass independently, thus allowing the true relationships to be inferred. Comparative studies of mammals *in vivo* and cell culture have previously allowed researchers to test hypotheses about how natural selection shapes aging. Recently two studies have focused on rodents to investigate the relationship between telomerase [19, 20], and transcription factors in the brain [21] with aging, body mass, and other life history traits. In order to test if IIS is correlated to either body mass or longevity across taxa, we used our tissue collection from sixteen rodent species and analyzed the IGF1R levels in four different organs (heart, lung, kidney and brain) by Western blot.

## RESULTS

We collected and dissected multiple adult specimens from sixteen rodent species (Table 1). The tissues were flash-frozen in liquid nitrogen immediately upon dissection. All of the collected individuals were young adults, although precise ages could not be established as most were wild-caught. Whole protein samples were prepared from heart, kidney, lung and brain sections. Sections from the brain were taken exclusively from the frontal cortex, with the olfactory bulb and the pituitary gland omitted. Protein samples were tested for total concentration via a Lowry assay and frozen at −80°C.

**Table 1:** Specimens used in this study. Maximum Lifespan and Adult Body Mass values were taken from the AnAge database [41]. Numbers under the organ headings indicate the number of independent protein samples analyzed from different individuals for each organ and species. All animals were adult when sacrificed, although their exact age could not be verified.

Common Name	Species Name	Maximum Lifespan (Years)	Adult Body Mass (g)	BRAIN	LUNG	HEART	KIDNEY
House Mouse	*Mus musculus*	4	30	3	3	3	2
Norway Rat	*Rattus norvegicus*	5	400	2	2	2	2
Red Squirrel	*Tamiascuirus hudsonicus*	10	200	2	3	2	2
American Beaver	*Castor canadensis*	24	20,250	3	2	3	2
Woodchuck	*Marmota monax*	14	5,000	2	2	3	2
Blind Mole-Rat	*Nannospalax ehrenbergi*	15	160	3	3	2	2
Eastern Grey Squirrel	*Sciurus carolinensis*	24	533	2	2	3	2
Golden Hamster	*Mesocricetus auratus*	4	105	2	2	2	2
Naked mole-rat	*Heterocephalus glaber*	31	35	2	2	2	2
Chinchilla	*Chinchilla lanigera*	17	642	3	2	3	2
Capybara	*Hydrochaeris hydrochaeris*	15	55,000	2	2	2	2
Paca	*Agouti paca*	16	9,000	2	2	2	2
Guinea Pig	*Cavia porcellus*	12	738	4	2	2	2
Fox Squirrel	*Sciurus niger*	16	800	3	2	2	2
Mongolian Gerbil	*Meriones unguiculatus*	6	53	2	0	0	0
Deer Mouse	*Peromyscus maniculatus*	8	20	2	2	2	2

### The IGF1R Epitope Region is Highly Conserved Across Fourteen Rodent Species

Because we wished to use Western blotting as a quantitative assay of protein concentration it was necessary to control for potential differential binding of the antibody to different amino acid sequences between species.

**Figure 1:**
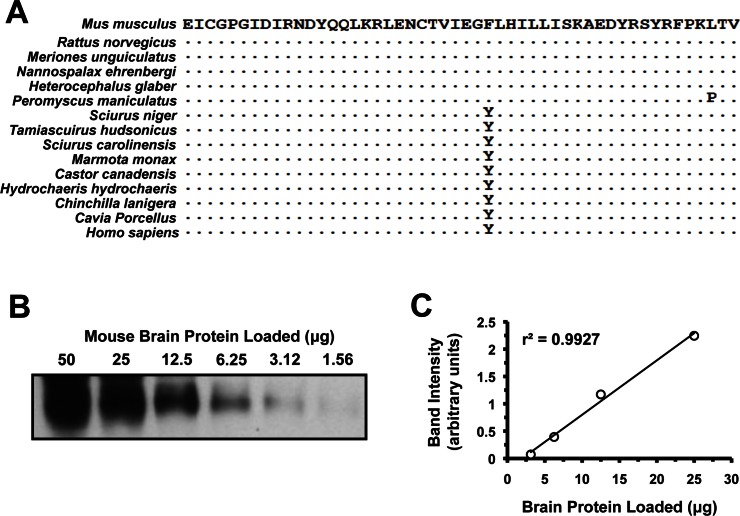
Quantitative analysis of IGF1R protein levels. (**A**) Alignment of the 50 amino acids comprising the epitope recognized by the anti-IGF1R polyclonal antibody used (SC-712, see Experimental Procedures). This region maps to the N-terminus of the protein, which is on the alpha-chain peptide of IGF1R. It excludes the signaling peptide, which is cleaved during protein maturation, and was not used in the immunogen. Human sequence is included due to it being used to generate the antibody. Sequence identity is indicated by dots. (**B**) Western blot of serial dilutions of mouse brain whole tissue protein extracts. Linear region was between 25 and 3.12 μg. (**C**) Band intensity in arbitrary units, plotted against protein loaded to verify linearity.

In order to confirm sufficient conservation for cross-species analysis, we performed RT-PCR using primers to the 5' end of IGF1R alpha subunit prior to the protein coding sequence and 3' primers sufficiently downstream to encompass the epitope site. Paca and hamster mRNAs could not be amplified, but for all other species the products were cloned, sequenced, translated *in silico* and aligned. (Figure 1A). Between species, the IGF1R alpha subunit epitope was very well conserved. Only the deer mouse *P. maniculatus* had a significantly divergent amino acid but it did not seem to interfere with antibody reactivity; otherwise the sequences divided into two variants (tyrosine or phenylalanine at position 28). Quantitative inspection after Western blot analysis did not reveal a statistically significant signal bias towards either variant.

**Figure 2:**
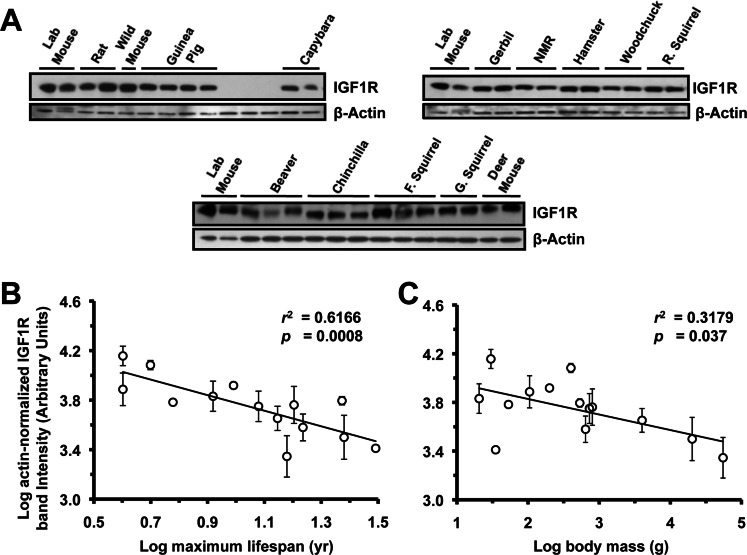
Levels of IGF1R protein in brain tissue are highly negatively correlated to lifespan, and weakly negatively correlated to body mass. (**A**) Western blots showing IGF1R and actin bands. (**B**) Log-transformed graph of IGF1R intensity plotted against maximum lifespan shows strong correlation (r^2^=0.61, p=0.0008). (**C**) Log-transformed graph of IGF1R intensity plotted against average adult body mass show marginally significant correlation (r^2^=0.31, p=0.037). Error bars: 1 SD. Correlation of brain IGF1R to lifespan was still strong (*r*^2^ = 0.58, *p* = 0.0009) after multiple regression analysis factoring in the contribution of body mass to lifespan, whereas correlation between body mass and lifespan was non-significant (adjusted *r*^2^ = 0.14, *p*=0.1). Phylogenetic correction by independent contrasts maintained a significant correlation to lifespan (*r*^2^= 0.374, *p*= 0.0261) while the correlation to body mass was rendered non-significant (*r*^2^= 0.189, *p*= 0.136).

In order to ensure that Western blot data would be quantitative, we performed serial dilutions of mouse brain extracts and quantified them (Figure 2B and 2C). The antibody (rabbit polyclonal targeting the N-terminus of IGF-IRα, see Experimental Procedures) was found to respond in a linear fashion to protein quantity as long as the band was not over-saturated or only barely visible. Various actin antibodies were also tested for use as appropriate loading controls. β-actin is highly conserved across all metazoan taxa, and given the perfect conservation between human, mouse and chicken we did not sequence it for analysis in our rodent species.

### Level of IGF1R Protein in Rodent Brain Tissue is Strongly Negatively Correlated to Lifespan

Because published data from other model organisms strongly suggested the involvement of the nervous system in IGF1R mediated lifespan modulation [22, 23], we started by performing Western blots to quantify the levels of IGF1R in rodent brains (Figure 1A). For unknown reasons, *Nannospalax* individuals did not react with the antibody (despite having identical sequence to mouse) and thus were omitted from analysis. IGF1R intensity showed a significant negative correlation to maximum lifespan (*r*^2^ = 0.6166, *p* = 0.0008) (Figure 1B). IGF1R was also correlated to body mass, albeit weakly (*r*^2^ = 0.3179, *p* = 0.037) (Figure 2C). Body mass was previously shown to correlate positively with longevity across species [24-26]. To control for the effects of possible correlations between body mass and lifespan, we analyzed the independent contribution of each by multiple regression. Multiple regression analysis showed that only maximum lifespan was significantly related to IGF1R levels (*F*_2,11_ = 11.2, *p* = 0.002, maximum lifespan, *t* = −3.45, *p* = 0.005; body mass, *t* = −1.31, *p* = 0.21).

Because the rodent species are related to each other, they cannot be considered independent data points. In order to account for the common descent of the various rodents, we analyzed phylogenetically independent contrasts [27]. As many of the short-lived species were from the family Muridae, the correlation of IGF1R levels in the brain to lifespan was weakened but remained significant (*r*^2^ = 0.369, *p*= 0.027) while the correlation of IGF1R levels in the brain to body mass was rendered insignificant (*r*^2^ = 0.26, *p* = 0.074). Collectively, the IGF1R expression level in the brain was strongly negatively correlated to longevity, and this negative correlation persisted after adjustments for the body mass-lifespan correlation, and phylogenetic non-independence.

### IGF1R Levels in Lung, Heart, and Kidney Are Not Correlated to Lifespan or Body Mass

**Figure 3:**
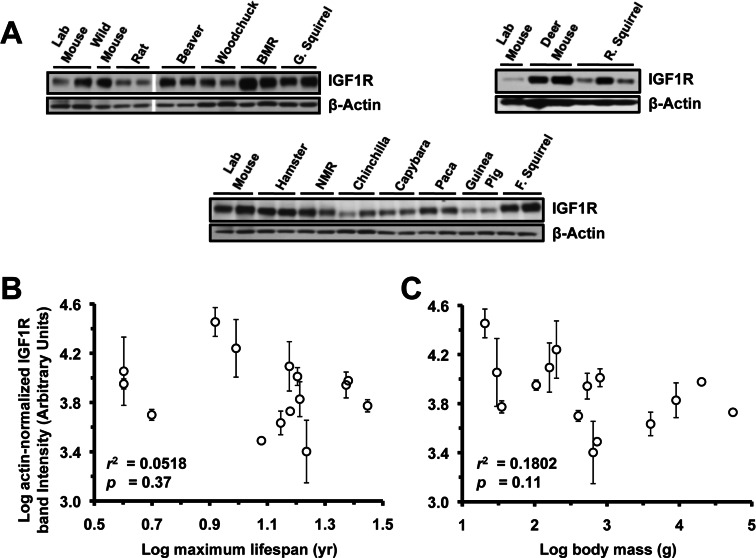
Levels of IGF1R protein in lung tissue do not correlate with lifespan or body mass. (**A**) Western blots showing IGF1R and actin bands. (**B**) Log-transformed graph of IGF1R intensity plotted against maximum lifespan shows very weak (non-significant) correlation (r^2^=0.05). (**C**) Log-transformed graph of IGF1R intensity plotted against average adult body mass show very weak (non-significant) correlation (r^2^=0.18, p=0.11). Error bars are s.d.

**Figure 4:**
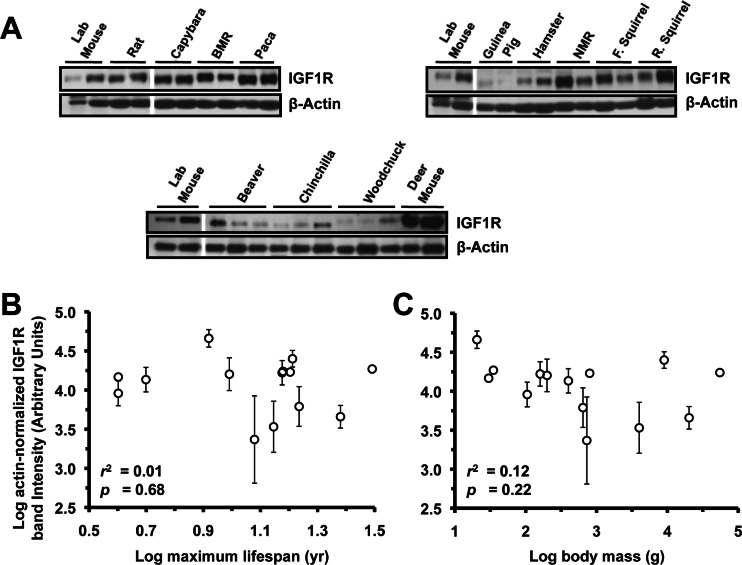
Levels of IGF1R protein in heart tissue do not correlate with lifespan or body mass. (**A**) Western blots showing IGF1R and actin bands. (**B**) Log-transformed graph of IGF1R intensity plotted against maximum lifespan shows no correlation. (**C**) Log-transformed graph of IGF1R intensity plotted against average adult body mass shows no correlation. Error bars are s.d.

**Figure 5:**
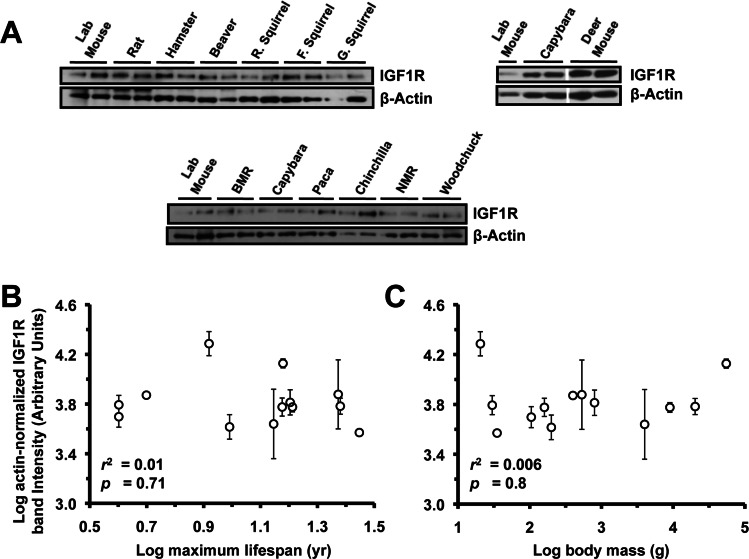
Levels of IGF1R protein in kidney tissue do not correlate with lifespan or body mass. (**A**) Western blots showing IGF1R and actin bands. (**B**) Log-transformed graph of IGF1R intensity plotted against maximum lifespan shows no correlation. (**C**) Log-transformed graph of IGF1R intensity plotted against average adult body mass shows no correlation. Error bars are s.d.

Extracts from lung (Figure 3A), heart (Figure 4A), and kidney (Figure 5A) were also assayed by Western blot for levels of IGF1R. None of the organs showed significant correlation to lifespan (Figure 3B, 4B, 5B) or body mass (Figure 3C, 4C, 5C). No correlation was found for the raw data and no correlation was found after phylogenetic correction by independent contrasts. In summary, only brain IGF1R levels showed correlation to lifespan.

## DISCUSSION

We have analyzed the levels of the IGF1R protein in the brain, lung, heart, and kidney tissues of 16 rodent species. We found that IGF1R levels are highly variable across species, and only correlate to lifespan in the brain tissue. The correlation was negative, meaning that longer-lived species had lower IGF1R levels in the brain, irrespective of body mass. Hence, even larger-bodied, long-lived species such as beaver expressed lower IGF1R levels in the brain. This finding agrees with earlier studies in model organisms where reduced IIS was associated with longevity. Furthermore, our results indicate that small body mass observed in IIS mutants may be a secondary consequence of artificially lowered IIS signaling, while on the evolutionary scale only IIS signaling in the brain contributes to longevity, whereas the body mass and IIS in peripheral tissues vary independently.

The finding that IGF1R levels in the brain are negatively correlated to lifespan, but not to body mass, may help resolve the paradox of species body mass and aging. Within species, body mass is *inversely* correlated to lifespan, and the differences in body mass are sometimes driven by the IIS [17]. Across species, however, body mass is positively correlated to lifespan, and it is not clear what drives this. Specifically how evolutionary forces shape IIS activity is unclear. One possible selective pressure that might increase IGF1R levels in the brain is a need to speed up development due to environmental or predatory constraints. This may lead to decreased lifespan due to increased activity of nutrient and growth factor-sensing pathways like mTOR. Recent evidence shows that male mice that have higher activation of mTOR and pAKT at 6 months of age, a time in their development when their body mass increases substantially (28%) when compared to females [28]. This, in turn, may explain why male mice have shorter lifespans than female mice. Conversely, lower extrinsic mortality experienced by large species can drive the evolution of slower developmental rate, lower IIS signaling and longer lifespan. How is then lower IIS compatible with larger body mass? Our results show that to achieve longevity IIS needs to be low in the brain only, but not in the peripheral tissues allowing animals to grow large while keeping developmental rates low.

Earlier studies using model organisms support the notion that IIS signaling in the nervous system is particularly important in the context of aging. Removal of IIS from neurons is sufficient to extend lifespan by the same amount as a whole-worm knockout [29]. The contribution of this pathway in the nervous system towards a systemic aging effect was also partially demonstrated in *Drosophila*, where activation of FOXO in the pericerebral fat body increased lifespan [30]. A brain-specific knock-out strategy in mice found that when IGF1R is heterozygously removed in the developing brain (causing a 50% reduction in total receptor), there is an extension of average lifespan in both males and females [22]. Most recently, reduction of IGF1R signaling by an miRNA in the brain of mutant mice was also found to increase their lifespan [31]. Another study on neural miRNAs has found that a group that includes IGF1R as a regulatory target affects proliferation and differentiation of neural progenitors [32].

Our study establishes an association between IGF1R levels in the brain and maximum lifespan in across rodent species. The correlation between lifespan and IGF1R was maintained even when adjusting for the contribution of body mass, and when adjusting for phylogenetic relatedness between rodents. A conservative estimate is that differences of IGF1R expression in brain tissue accounts for about 20% of lifespan differences observed between rodents. There was approximately a 10-fold difference in intensities between highest and lowest IGF1R signals in the rodent brain. Given that a 50% reduction in protein expression was sufficient to observe lifespan effects in mice [22] the 10-fold difference is likely to contribute to lifespan differences *in vivo* between rodent species.

Exactly how, mechanistically, the IGF1R receptor modulates lifespan through the nervous system is still a mystery. Although aging is generally seen as a phenomenon that affects all organ systems, it is possible that degeneration specifically of nervous system organization as the animal ages leads to wider systemic effects that lead to increased mortality. In *C. elegans*, inhibition of the *daf-2* signaling pathway prevented the formation of ectopic neurite branches that normally appear as a result of aging [33] and ablation of IIS in neurons is sufficient to extend lifespan [34]. In *Drosophila*, overexpression of antioxidant genes such as *SOD1* in motorneurons is sufficient to increase lifespan, possibly indicating that ROS damage in the nervous system can be a major lifespan determinant in insects [35]. In mammals, a growth factor that promotes neural survival was recently found to correlate to maximum rodent lifespan, also independently of phylogeny [21]. These results taken together with our data suggest that nervous system aging is important to overall longevity.

To date, there are no studies looking at the incidence of neurodegeneration of any kind in IGF1R whole body, brain-specific, or somatotroph specific knock-out mice. A very recent study demonstrated that deviations in the circadian rhythm of mice can predict lifespan [36]. This shows that variations in nervous system function can have systemic consequences leading to shortened lifespan.

Uncontrolled IGF1R signaling is an important hallmark of certain cancers and interfering with IGF1R signaling can help control malignancies [37]. If high IGF1R expression is limiting lifespan by induction of tumorigenesis, one might expect IGF1R levels to be inversely correlated with lifespan in all organs that have a high incidence of neoplasia. The lack of correlation we observed between IGF1R levels and lifespan in the kidney and lung suggests that the receptor does not act as a limit to lifespan by promoting tumorigenesis. High receptor expression may promote tumors in some species, but the maximum lifespan is not inherently limited by this risk.

In summary, our study supports previous findings that lowered IGF1R activity is correlated to extended lifespan in mammals, and expands this paradigm to interspecies comparisons. Furthermore, we show that on the evolutionary scale IIS signaling in brain tissue only influences lifespan, and this relationship is independent of body mass. These findings benefit our general understanding of the evolution of aging, and can be useful in designing potential life extending interventions where IIS signaling could be targeted in specific brain regions only avoiding undesirable severe side effects on peripheral tissues.

## METHODS

### RT-PCR and Sequence Alignment

RNA was extracted from rodent tissues by freezing in liquid nitrogen and pulverizing with a mortar and pestle, then running the homogenized tissue through a Qiagen RNEasy column. RNA was used for a RT-PCR reaction using a Titan One-Step Kit (Roche) Primer sequences were as follows: IGF1R F primer: 5' GAG AAA AGG GAA TTT CGT CCC AAA TAA AAG G- 3'. IGF1R R primer: 5' CTA TGG TGG AGA GGT AAC AGA GGT C- 3'. Band were cut and purified by Qiagen Qiaex resin and cloned into Invitrogen TOPO TA cloning kit. Plasmids were prepped from overnight culture of transformants and sent for sequencing at the University of Rochester Functional Genomics center. Sequences were imported into BioEdit [38] translated *in silico* then aligned manually.

### Protein Extraction

Approximately 100 milligrams of tissue were cut on dry ice. The sliver of tissue was then placed in a sterile Eppendorf tube, and homogenized on ice in RIPA buffer containing protease inhibitors. An equal volume of Laemlli buffer containing beta-mercaptoethanol was added, and the sample was boiled for 10 minutes with brief vortexing at the 5 minute mark. For samples used to measure phosphor-AKT, a cocktail of phosphatase inhibitors was added immediately after homogenization (Halt™ Phosphatase Inhibitor Cocktail, Thermo Scientific).

### Protein Quantification

Several microliters of protein samples were carefully diluted 10-fold and run through the Lowry assay per the manufacturer's instructions (Bio-Rad RC DC Protein Assay).

### Western Blot

Bio-Rad premade Criterion™ 4-15% SDS-polyacrylamide Tris-HCl gradient gels were loaded with protein samples and prestained markers. Mouse samples were loaded on every gel for cross-gel loading control. Proteins were transferred to nitrocellulose membranes using a Bio-Rad Trans-Blot Turbo transfer cassette. Membranes were blocked by incubating in 5% TBST-MILK for one hour, cut, and stained with primary antibodies to IGF1R or beta-actin overnight. Membranes were then washed, incubated with secondary antibodies for 1 hour, washed again, and developed after using GE Healthcare ECL. Antibodies used were: Santa Cruz (sc-712) Rabbit Polyclonal to IGF1R alpha chain, Santa Cruz (47778) Mouse Monoclonal to β-Actin, (Cell Signaling (#9271).

### Band Quantification and Data Analysis

Developed film was scanned and the resulting .TIFF images were imported into the Bio-Rad Quantity One software. Bands were selected using the Volume Rectangle Tool, and the ‘Adjusted Volume Intensity mm^2^ value' was used as an arbitrary unit of intensity. IGF1R Bands were normalized to actin based on the assumption that actin levels would be similar in the same tissue across species. Intensity was also normalized to mouse IGF1R across different gels (this adjustment was usually very small) due to differences in transfer efficiency, exposure, picture brightness. The mean IGF1R values for each tissue are shown in Supplemental Table 1. All data was log transformed for the statistical analysis. Normal distribution of IGF1R data was tested by Chi-Squared test for IGF1R band intensity in the brain (χ^2^ = 1.02, *p* = 0.6), rodent adult body mass (χ^2^ = 2.08, *p* = 0.35), and rodent maximum lifespan (χ^2^ = 1.58, *p* = 0.45). Normality was also verified via Q-Q plot analysis. Multiple regression analysis was performed using StatTools for Excel. Analysis of independent contrasts was performed using an implementation of CAIC algorithm [39] in MATLAB. The phylogenetic tree for independent contrasts was derived from data published in Meredith et al. [40] (Supplemental Figure 1). Equal branch lengths were assumed for the analysis.

## SUPPLEMENTARY INFORMATION


