# Supplementary material for: IGF1R levels in the brain negatively correlate with longevity in 16 rodent species
Source: Aging (Albany NY). 2013 Apr 25;5(4):304–14. doi: 10.18632/aging.100552 (PMC3651522; doi:10.18632/aging.100552)
Supplement: Supplementary file 1 [file aging-05-304-s001.pdf]

38. Hall TA. BioEdit: a user-friendly biological sequence alignment editor and analysis program for Windows 95/98/NT. Nucl Acids Symp Ser. 1999; 41:95-98.
39. Purvis A and Rambaut A. Comparative analysis by independent contrast (CAIC): an Apple Macintosh application for analysing comparative data. Comp Appl Biosci. 1995; 11:247-251.
40. Meredith RW, Janecka JE, Gatesy J, Ryder OA, Fisher CA, Teeling EC, Goodbla A, Eizirik E, Simao TL, Stadler T, Rabosky DL, Honeycutt RL, Flynn JJ, et al. Impacts of the Cretaceous Terrestrial Revolution and KPg extinction on mammal diversification. Science. 2011; 334:521-524.
41. de Magalhaes JP, and Costa, J. . A database of vertebrate longevity records and their relation to other life-history traits. Journal of Evolutionary Biology 2009; 22:1770-1774.

SUPPLEMENTARY INFORMATION

Supplemental Table 1. Band intensity values for IGF1R immunoblots and the corresponding standard deviation for each tissue assayed. Intensity values were adjusted for β-actin intensity differences as well as gel intensity differences (mouse samples were loaded into each gel as a control). Samples omitted from analysis are indicated by a dash.

	Brain		Lung		Heart		Kidney	
**Species**	**IGF1R**	**SD**	**IGF1R**	**SD**	**IGF1R**	**SD**	**IGF1R**	**SD**
Mus musculus	14358	2680	11324	4618	14690	1382	6220	1086
Rattus norvegicus	12106	1023	5005	508	13601	4787	7438	66
Cavia porcellus	5620	1623	3088	89	2335	2259	-	-
Hydrochaeris hydrochaeris	2214	832	5357	15	17342	1587	13409	1117
Meriones unguiculatus	6064	45	-	-	-	-	-	-
Heterocephalus glaber	2575	66	5934	676	18591	112	3718	69
Mesocricetus auratus	7710	2305	8928	839	9089	3220	4987	963
Marmota monax	4493	1000	4304	943	3393	2698	4362	2630
Tamiascuirus hudsonicus	8286	430	17389	9302	15959	7325	4129	931
Sciurus niger	5771	1119	10257	1708	16989	192	6506	1505
Sciurus carolinensis	6238	939	8757	2100	-	-	7548	4528
Castor canadensis	3158	1995	9488	390	4566	1383	6065	900
Chinchilla lanigera	3797	485	2521	1396	6164	3564	-	-
Peromyscus maniculatus	6781	1875	28454	7578	45825	11649	19340	4297
Nannospalax ehrenbergi	-	-	12417	5542	16646	5867	5971	997
Agouti paca	-	-	6714	2151	25118	6074	5966	531

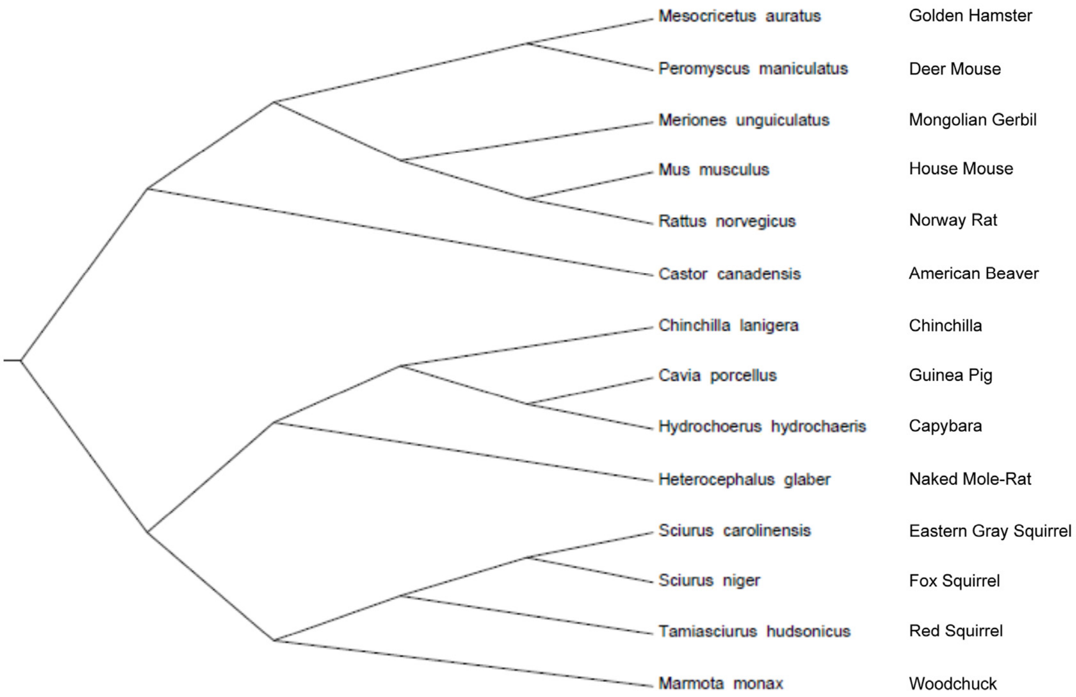

Supplemental Figure 1. Phylogenetic tree topology used for independent contrast analysis. The tree topology was inferred from Meredith et al. Science 2011 334:521-4 [40] and NCBI taxonomy information. Branch lengths were set as equal.
